# Real-world outcomes of multimodal treatment for pleural mesothelioma: A retrospective study in a high-volume integrated regional center

**DOI:** 10.1016/j.xjon.2026.101603

**Published:** 2026-01-30

**Authors:** Alison S. Baskin, Keza Levine, Yun-Yi Hung, Elaine Liang, Kian C. Banks, J. Marie Suga, Jeffrey B. Velotta

**Affiliations:** a Department of Surgery, UCSF, San Francisco, Calif; b School of Medicine, UCSF, San Francisco, Calif; c Division of Research, Kaiser Permanente Northern California, Oakland, Calif; d Kaiser Permanente Bernard J. Tyson School of Medicine, Pasadena, Calif; e Department of Surgery, UCSF East Bay, Oakland, Calif; f Department of Oncology, Kaiser Vallejo Medical Center, Vallejo, Calif; g Division of Thoracic Surgery, Department of Surgery, Kaiser Permanente Oakland, Oakland, Calif

**Keywords:** pleural mesothelioma (PM), multimodal therapy, pleurectomy, decortication, epithelioid, early-stage

## Abstract

**Objective:**

To evaluate overall survival (OS) associated with multimodal treatment regimens, including pleurectomy/decortication (PD) and systemic therapy, in patients with clinical stage I-II epithelioid pleural mesothelioma (PM).

**Methods:**

We identified patients with PM treated within an integrated health care system (2009–2023). We grouped patients by treatment: no treatment, surgery, systemic therapy, and multimodal treatment (surgery + systemic therapy). The primary outcome was median OS. Using multivariable Cox regression, we assessed associations between treatment and OS, adjusting for clinical characteristics. Subgroup analyses were performed for patients with clinical stage I-II epithelioid PM.

**Results:**

Among all eligible patients (n = 432), those who received no treatment (n = 198), surgery (n = 25), systemic therapy (n = 164), and multimodal treatment (n = 45) had median OS of 5 months (95% CI, 4-7), 11 (6-17), 13 (12-17), and 27 (20 to nonestimable [NE]) (*P* < .0001). Among patients with clinical stage I-II epithelioid PM (n = 112), those who received no treatment (n = 40), surgery (n = 10), systemic therapy (n = 42), and multimodal treatment (n = 20) had median OS of 10 (7-17), 29 (5-NE), 18 (17-26), and 37 (25-NE) months (*P* < .0005). Adjusted analyses showed improved OS relative to no treatment with multimodal treatment but not systemic therapy in the clinical stage I-II epithelioid subgroup (multimodal therapy: adjusted hazard ratio, 0.35; 95% CI, 0.15-0.85, *P* = .02; systemic therapy: adjusted hazard ratio, 0.66; 95% CI, 0.37-1.16, *P* = .15).

**Conclusions:**

In a real-word cohort of patients with early-stage epithelioid PM, multimodal treatment incorporating surgery and systemic therapy was associated with improved OS compared with no treatment. These findings reinforce the benefit of surgery in carefully selected patients, despite recent studies questioning its role in a broader population.

## Graphical Abstract

**Figure:**
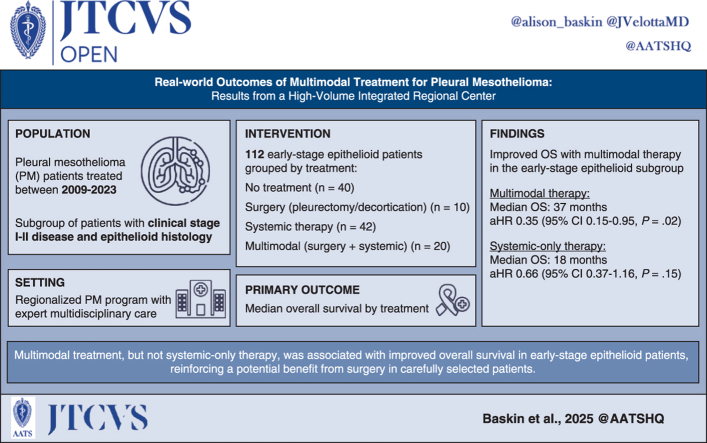

**Figure:**
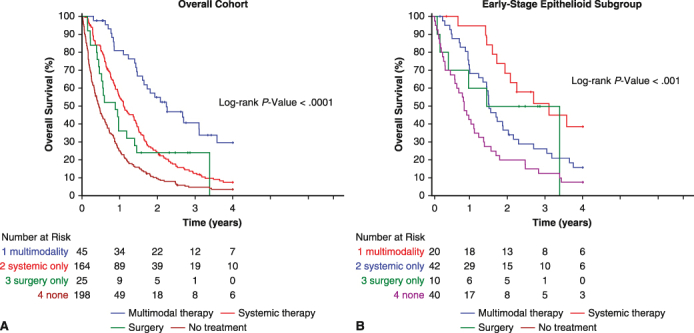
Does surgery in a multimodal treatment regimen improve survival for pleural mesothelioma?

**Central Message**

Surgery for pleural mesothelioma remains controversial. Pleurectomy/decortication as part of multimodal therapy improves overall survival in patients with early-stage epithelioid mesothelioma.

**Perspective**

It is increasingly important to study the role of surgery among carefully selected patients with pleural mesothelioma (PM) in a real-world setting. In this contemporary analysis from a large integrated health care system, we evaluated overall survival associated with pleurectomy/decortication in a multimodal regimen for patients with early-stage epithelioid PM.

Pleural mesothelioma (PM) is characterized by aggressive clinical behavior and a poor prognosis. The 3 predominant histologic subtypes, epithelioid, sarcomatoid, and biphasic, differ significantly in both prognosis and therapeutic response.^1^^,^^2^ Most patients are diagnosed at advanced stages, making treatment especially challenging. Available treatment modalities include systemic chemotherapy, surgery, radiation therapy, and immunotherapy. Among these, surgery remains the most controversial.^3^, ^4^, ^5^, ^6^

Numerous studies have evaluated whether surgery improves survival in PM, yielding mixed results. Retrospective analyses have suggested associations between surgery and longer survival.^7^, ^8^, ^9^, ^10^ In contrast, the 2011 Mesothelioma and Radical Surgery (MARS) feasibility trial suggested that radical surgery, specifically extrapleural pneumonectomy (EPP), not only fails to improve outcomes but also causes harm.^11^ Its successor trial, MARS2, similarly demonstrated no benefit from extended pleurectomy and decortication (PD) after neoadjuvant chemotherapy.^12^ MARS2 has not only sustained the debate over the role of surgery in PM but also has brought patient selection to the forefront, largely attributable to aspects of its study design, including broad inclusion criteria, limited preoperative staging, and randomization after neoadjuvant chemotherapy.^6^^,^^13^

Current clinical guidelines emphasize the importance of careful patient selection. The 2025 American Society of Clinical Oncology guidelines recommend surgery with systemic therapy for highly selected patients with favorable prognostic characteristics, such as early-stage epithelioid tumors, while discouraging maximal surgical cytoreduction for patients with the sarcomatoid subtype.^14^ Similarly, the National Comprehensive Cancer Network guidelines advise surgical resection as part of multimodal therapy for medically operable patients with stage I-IIIA disease.^15^

Despite these recommendations, real-world evidence evaluating outcomes of lung-sparing surgery in patients with favorable features is lacking. To address this gap, we conducted a retrospective analysis within a large integrated health care system to evaluate outcomes associated with surgery as part of a multimodal treatment strategy. We hypothesized that PD surgery, in combination with systemic therapy, would improve survival in patients with early-stage epithelioid PM.

## Methods

### Approval and Reporting

This multicenter, retrospective study was granted a waiver of informed consent by the Kaiser Permanente Northern California (KPNC) Institutional Review Board (#1714772) on May 2, 2025. We report this study in accordance with the Strengthening the Reporting of Observational Studies in Epidemiology checklist.

### Study Population and Setting

Through review of electronic medical records, we identified patients aged ≥18 years at diagnosis with histologically confirmed PM, treated within KPNC between January 1, 2009, and December 31, 2023. KPNC is a large integrated health care system with 21 medical centers, serving approximately 4.4 million patients, or 33% of northern California's population.^16^ KPNC regionalized its PM surgical services to 4 hospitals in 2014 and introduced a regional weekly bi-disciplinary tumor board with medical oncology and thoracic surgery in 2017.

Patients in this study were staged with computed tomography and positron emission tomography scans, followed by mediastinoscopy if indicated. We excluded patients who underwent EPP, restricting surgical patients to those who underwent PD.^17^ Two high-volume surgeons performed all operations. Biologic mesh was used for diaphragm reconstruction when necessary.^18^ Intrapleural betadine scrub was used as an adjunctive treatment during all surgeries. Patients who received both surgery and systemic treatment received chemotherapy after surgery.

### Data Sources and Variables

We used data from the KPNC cancer registry (meeting Surveillance, Epidemiology, and End-Results standards) and HealthConnect, Kaiser Permanente's EPIC-based electronic health record. Using both databases, we extracted patient-level data on diagnosis date, age, sex, race/ethnicity, Charlson Comorbidity Index (CCI) score, smoking history, histology, clinical stage, surgery date, and postoperative complications. Major complications included acute respiratory failure, cardiac arrest, or death within 30 days.

The primary outcome was median overall survival (OS), defined as time from cancer diagnosis date to date of death (any cause), KPNC health plan discontinuation, or last follow-up (through December 31, 2024), whichever occurred first. Secondary outcomes included 24-month survival, 30-day postoperative complications, 30-day major postoperative complications, and 30- and 90-day mortality.

### Statistical Analysis

**Figure E1:**
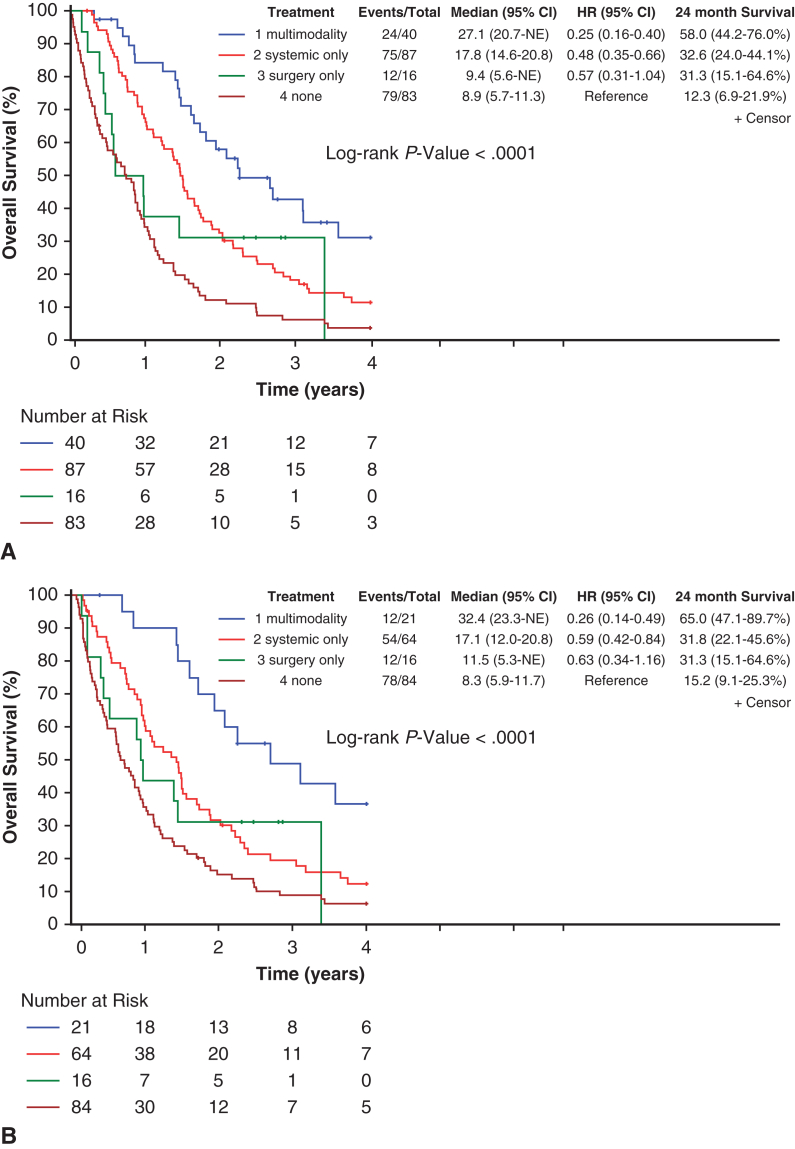
Unadjusted Kaplan-Meier curves by treatment type for the epithelioid subgroup (A) and early-stage subgroup (B). Corresponding 95% CIs are provided in the accompanying table. *HR*, Hazard ratio.

**Figure E2:**
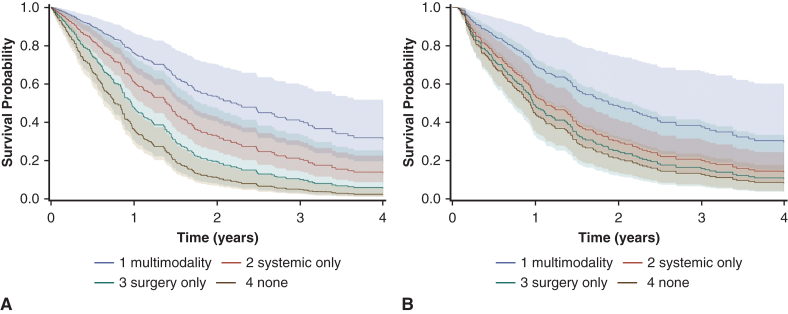
Adjusted survival curves by treatment for the epithelioid subgroup (A) and early-stage subgroup (B). *Shaded areas* represent 95% CIs.

Patients were grouped by treatment: no treatment, surgery without systemic therapy (PD), systemic therapy without surgery, and multimodal treatment (surgery and systemic therapy). Patients in the surgery, systemic therapy, and multimodal treatment groups may have also received radiation therapy. Baseline characteristics were compared across treatment groups using the χ^2^, Fisher exact, or analysis of variance tests. We performed a subgroup analysis of patients who had early-stage disease (clinical stage I-II) and epithelioid histology. Additional analyses of patients with early-stage disease (across all histologies) and those with epithelioid histology (across all stages) are provided in Table E1, Table E2, Table E3, Table E4 and Figures E1 and E2.

The Kaplan-Meier method was used to estimate unadjusted survival, and log-rank tests were used to compare the survival curves across treatment groups. Multivariable Cox proportional hazards models assessed associations between treatment regimen and OS, adjusting for diagnosis period (2009-2014, 2015-2020, and 2021-2023), age at diagnosis, sex, histology and/or stage (as appropriate), and CCI. Analyses were performed using SAS, version 9.4.

## Results

### Treatment Patterns and Patient Characteristics

A total of 438 patients were identified. Six patients underwent EPP and were excluded. Among the remaining 432 patients, 198 (45.8%) received no treatment, 25 (5.8%) underwent PD surgery, 164 (38.0%) received systemic therapy, and 45 (10.4%) received multimodal therapy (Table 1). In patients with early-stage epithelioid PM (n = 112), these proportions were 35.7% (no treatment), 8.9% (surgery), 37.5% (systemic therapy), and 17.9% (multimodal therapy) (Table 2). Baseline characteristics were similar across treatment groups except for age and diagnosis year in both cohorts, as well as histology and clinical stage in the overall cohort.

**Table 1:** Patient characteristics of the entire cohort by treatment (N = 432)

Characteristic	No treatment (n = 198)	Surgery alone (n = 25)	Systemic-only (n = 164)	Multimodal (n = 45)	*P* value
Age at diagnosis, y					<.0001
Mean ± SD	79.2 ± 10.1	71.2 ± 10.7	72.5 ± 8.7	68.7 ± 10.0	
Median (IQR)	81.0 (75.0-86.0)	74.0 (70.0-77.0)	74.0 (67.0-78.0)	71.0 (65.0-74.0)	
CCI					.54
0-3	82 (41.4)	12 (48.0)	86 (52.4)	22 (48.9)	
4-6	63 (31.8)	6 (24.0)	44 (26.8)	12 (26.7)	
≥7	53 (26.8)	7 (28.0)	34 (20.7)	11 (24.4)	
Sex					.44
Male	143 (72.2)	20 (80.0)	126 (76.8)	30 (66.7)	
Female	55 (27.8)	5 (20.0)	38 (23.2)	15 (33.3)	
Race/ethnicity					.71
White	136 (68.7)	16 (64.0)	118 (72.0)	28 (62.2)	
Black	14 (7.1)	3 (12.0)	5 (3.0)	2 (4.4)	
Hispanic	26 (13.1)	5 (20.0)	24 (14.6)	8 (17.8)	
Asian/Pacific Islander	11 (5.6)	0	9 (5.5)	4 (8.9)	
Native American/multiracial/other/unknown	11 (5.6)	1 (4.0)	8 (4.9)	3 (6.7)	
Clinical stage					<.01
1	51 (25.8)	8 (32.0)	46 (28.0)	11 (24.4)	
2	33 (16.7)	8 (32.0)	18 (11.0)	10 (22.2)	
3	55 (27.8)	8 (32.0)	41 (25.0)	19 (42.2)	
4	59 (29.8)	1 (4.0)	59 (36.0)	5 (11.1)	
Histology					<.0001
Epithelioid	83 (41.9)	16 (64.0)	87 (53.0)	40 (88.9)	
Biphasic	25 (12.6)	6 (24.0)	18 (11.0)	5 (11.1)	
Sarcomatoid	34 (17.2)	1 (4.0)	21 (12.8)	0	
NOS	56 (28.3)	2 (8.0)	38 (23.2)	0	
Smoking status					.73
Current	7 (3.5)	1 (4.0)	7 (4.3)	1 (2.2)	
Former	111 (56.1)	13 (52.0)	80 (48.8)	20 (44.4)	
Never	80 (40.4)	11 (44.0)	77 (47.0)	24 (53.3)	
BMI					.16
<25	98 (49.5)	10 (40.0)	58 (35.4)	18 (40.0)	
25.0-29.9	70 (35.4)	11 (44.0)	79 (48.2)	17 (37.8)	
≥30	30 (15.2)	4 (16.0)	27 (16.5)	10 (22.2)	
Diagnosis year					<.0001
2009-2014	96 (48.5)	5 (20.0)	65 (39.6)	1 (2.2)	
2015-2020	73 (36.9)	13 (52.0)	80 (48.8)	29 (64.4)	
2021-2023	29 (14.6)	7 (28.0)	19 (11.6)	15 (33.3)	

*SD*, Standard deviation; *IQR*, interquartile range; *CCI*, Charlson Comorbidity Index; *NOS*, not otherwise specified; *BMI*, body mass index.

**Table 2:** Characteristics of patients with early-stage epithelioid pleural mesothelioma by treatment (n = 112)

Characteristics	No treatment (N = 40)	Surgery (N = 10)	Systemic (N = 42)	Multimodal (N = 20)	*P* value
Age at diagnosis, y					.001
Mean ± SD	78.2 ± 11.0	70.3 ± 12.2	71.0 ± 8.8	68.7 ± 8.4	
Median (IQR)	80.5 (75.5-86.0)	74.5 (70.0-78.0)	70.0 (65.0-78.0)	70.0 (63.0-74.0)	
CCI					.77
0-3	15 (37.5)	3 (30.0)	21 (50.0)	7 (35.0)	
4-6	11 (27.5)	3 (30.0)	12 (28.6)	6 (30.0)	
≥7	14 (35.0)	4 (40.0)	9 (21.4)	7 (35.0)	
Sex					.23
Male	26 (65.0)	6 (60.0)	34 (81.0)	12 (60.0)	
Female	14 (35.0)	4 (40.0)	8 (19.0)	8 (40.0)	
Race/ethnicity					.42
White	26 (65.0)	5 (50.0)	33 (78.6)	15 (75.0)	
Black	1 (2.5)	1 (10.0)	1 (2.4)		
Hispanic	7 (17.5)	3 (30.0)	3 (7.1)	2 (10.0)	
Asian/Pacific Islander	5 (12.5)		4 (9.5)	1 (5.0)	
Native American/multiracial/other/unknown	1 (2.5)	1 (10.0)	1 (2.4)	2 (10.0)	
Clinical stage					.41
1	24 (60.0)	6 (60.0)	30 (71.4)	10 (50.0)	
2	16 (40.0)	4 (40.0)	12 (28.6)	10 (50.0)	
Smoking status					.63
Current	2 (5.0)	0 (0.0)	4 (9.5)	0 (0.0)	
Former	22 (55.0)	5 (50.0)	22 (52.4)	9 (45.0)	
Never	16 (40.0)	5 (50.0)	16 (38.1)	11 (55.0)	
BMI					.83
<25	19 (47.5)	3 (30.0)	19 (45.2)	7 (35.0)	
25.0-29.9	13 (32.5)	4 (40.0)	17 (40.5)	8 (40.0)	
≥30	8 (20.0)	3 (30.0)	6 (14.3)	5 (25.0)	
Diagnosis year					<.0001
2009-2014	25 (62.5)	1 (10.0)	16 (38.1)	0 (0.0)	
2015-2020	11 (27.5)	5 (50.0)	22 (52.4)	16 (80.0)	
2021-2023	4 (10.0)	4 (40.0)	4 (9.5)	4 (20.0)	

*SD*, Standard deviation; *IQR*, interquartile range; *CCI*, Charlson Comorbidity Index; *BMI*, body mass index.

### Overall Survival

**Figure 1:**
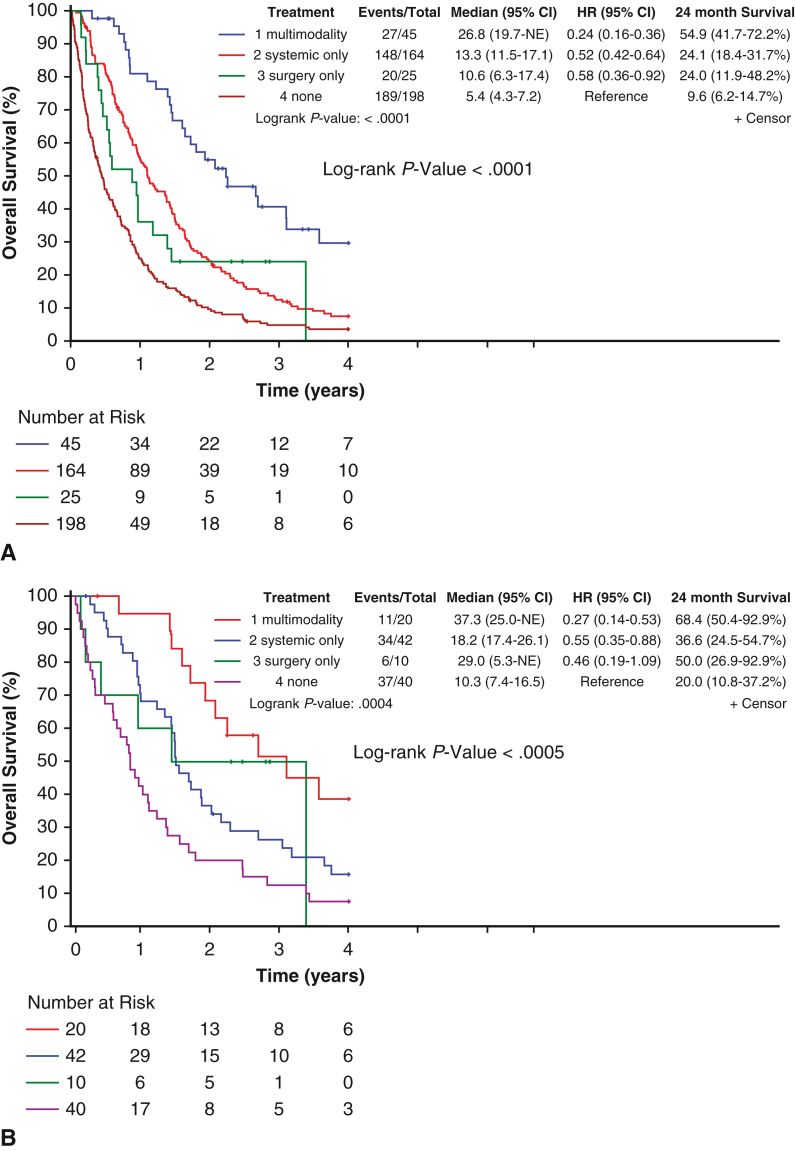
Unadjusted Kaplan-Meier survival curves by treatment type for the entire cohort (A) and early-stage epithelioid subgroup (B). Corresponding 95% CIs are provided in the accompanying table. *HR*, Hazard ratio.

The median OS across the entire cohort was 5.4 months (95% CI, 4.3-7.2 months) for no treatment, 10.6 months (6.3-17.4 months) for surgery, 13.3 months (11.5-17.1 months) for systemic therapy, and 26.8 months (19.7-nonestimable months) for multimodal therapy (*P* < .0001) (Figure 1). Median OS trends were similar in the early-stage epithelioid subgroup (10.3, 29.0, 18.2, and 37.3 months, *P* < .0005). Across both cohorts, 24-month survival rates were greatest among patients who received multimodal therapy (54.9 months in the overall cohort; 68.4 months in the early-stage epithelioid subgroup).

### Multivariable Cox Proportional Hazards Model

**Figure 2:**
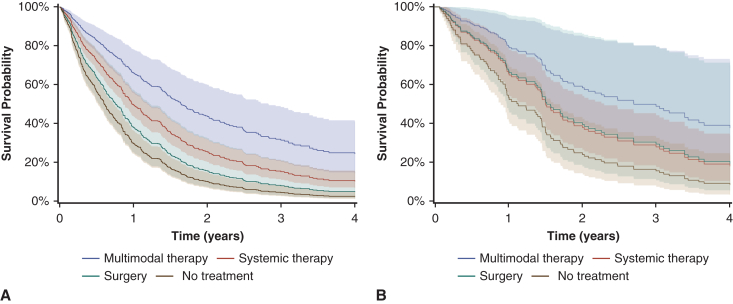
Adjusted survival curves by treatment type for the entire cohort (A) and early-stage epithelioid subgroup (B). *Shaded areas* represent 95% CIs.

After adjusting for diagnosis period, age, sex, histology, stage, and CCI, systemic therapy (adjusted hazard ratio [aHR], 0.56; 95% CI, 0.44-0.70, *P* < .001) and multimodal therapy (aHR, 0.32; 95% CI, 0.21-0.51, *P* < .001) remained significantly associated with improved OS in the entire cohort (Table 3 and Figure 2). However, in patients with early-stage epithelioid PM, multimodal therapy (aHR, 0.35; 95% CI, 0.15-0.85, *P* = .02), but not systemic therapy (aHR, 0.66; 95% CI, 0.37-1.16, *P* = .15), remained significant.

**Table 3:** Cox proportional hazards model across cohorts

Variable	Entire cohort		Early-stage epithelioid subgroup	
	aHR (95% CI)	*P* value	aHR (95% CI)	*P* value
Period				
2009-2014	Reference		Reference	
2015-2020	0.99 (0.78-1.26)	.96	0.70 (0.41-1.20)	.19
2021-2023	0.93 (0.65-1.33)	.69	0.43 (0.18-1.07)	.07
Age, y				
18-64	Reference		Reference	
65-74	1.26 (0.89-1.77)	.19	1.93 (0.98-3.81)	.06
75-84	1.50 (1.08-2.09)	.02	1.77 (0.89-3.52)	.10
≥85	1.76 (1.19-2.61)	.01	2.27 (0.98-5.22)	.05
Treatment				
No treatment	Reference		Reference	
Surgery	0.78 (0.48-1.27)	.32	0.63 (0.24-1.67)	.35
Systemic	0.56 (0.44-0.70)	<.001	0.66 (0.37-1.16)	.15
Multimodal	0.32 (0.21-0.51)	<.001	0.35 (0.15-0.85)	.02
Sex				
Female	Reference		Reference	
Male	1.10 (0.87-1.39)	.42	1.03 (0.63-1.69)	.90
CCI				
0-3	Reference		Reference	
4-6	0.89 (0.69-1.14)	.35	1.03 (0.59-1.80)	.93
≥7	1.04 (0.79-1.36)	.78	1.65 (0.95-2.87)	.08
Histology				
Non-epithelioid	Reference		–	
Epithelioid	0.68 (0.55-0.84)	<.001	–	
Stage				
I-II	0.60 (0.49-0.75)	<.001	–	
III-IV	Reference		–	

*aHR*, Adjusted hazards ratio; *CCI*, Charlson Comorbidity Index.

### Short-Term Surgical Outcomes

Among all surgical patients, 30-day complication rates were 54.3% for the entire cohort and 43.3% for early-stage epithelioid patients, whereas 30-day major complication rates were 10.0% for each cohort, respectively (Table 4). Thirty-day mortality rates were 7.1% (overall) and 6.7% (early-stage epithelioid), whereas 90-day mortality rates were 12.9% and 10.0%.

**Table 4:** Postoperative outcomes across cohorts

Outcomes	Entire cohort (N = 70)	Early-stage epithelioid subgroup (N = 56)
30-d emergency department visit	17 (24.3)	6 (20.0)
30-d readmission	10 (14.3)	4 (13.3)
30-d postoperative complication∗	38 (54.3)	13 (43.3)
30-d major postoperative complication†	7 (10.0)	3 (10.0)
30-d mortality	5 (7.1)	2 (6.7)
90-d mortality	9 (12.9)	3 (10.0)

∗ Postoperative complications included anemia, chyle leak, hypotension, pleural effusion, wound infection, pneumonia, empyema, pulmonary embolus/deep vein thrombosis, pericardial effusion, atrial fibrillation, prolonged air leak, bronchopleural fistula, prolonged intubation, reintubation, tracheostomy, prolonged supplemental oxygen, mucous plug, atelectasis, urinary retention, urinary tract infection, stroke, acute respiratory failure, cardiac arrest, and deep vein thrombosis.

† Major complication included acute respiratory failure, cardiac arrest, or death within 30 days.

## Discussion

MARS2 heightened the debate on the optimal treatment strategy for patients with PM, making it more important than ever before to understand the impact of surgery on carefully selected patients.^12^ In this multicenter study of 432 patients with PM, we assessed the role of PD specifically in patients with early-stage epithelioid disease. Our findings suggest a potential survival benefit from a multimodal treatment regimen that includes surgery. This is consistent with previous retrospective studies^7^, ^8^, ^9^, ^10^^,^^12^ but in contrast to MARS2 and the recent National Cancer Database analysis by Zhan and colleagues.^19^ To our knowledge, this is the first real-world analysis in the post-MARS2 era that directly addresses these concerns and provides contemporary evidence supporting consideration of PD with adjuvant chemotherapy in appropriately staged, favorable-risk patients.

Recently, several MARS2 investigators retrospectively analyzed patients in the surgical arm of their original study.^13^ Responding to critiques of broad inclusion criteria and under staging, Waller and colleagues^13^ restaged and isolated patients with stage I-II epithelioid PM. They found a median OS of 32 months, which is similar to the 37-month OS observed in our early-stage epithelioid subgroup. As described by the authors, this survival is markedly greater than observed in the T1-2N0 epithelioid subgroup of MARS2, likely due to under staging.

Although it is tempting to draw comparisons between our study and MARS2, such comparisons should be made with caution, given substantial differences in study protocols and patient cohorts. In MARS2, patients were randomized to surgery or no surgery only after 2 cycles of neoadjuvant chemotherapy, likely introducing selection bias by requiring that individuals first demonstrate an ability to tolerate chemotherapy. In contrast, all patients in our multimodal cohort underwent surgery upfront in accordance with our institution's protocol, which is an approach that may reduce preoperative functional declines due to neoadjuvant therapy and is also supported by the absence of clear benefit from a neoadjuvant sequence.^20^

Over the past several decades, the surgical approach to PM has shifted toward less-morbid strategies. Multiple studies have shown reduced morbidity and mortality with PD compared with EPP, leading guidelines to favor parenchymal-sparing operations.^21^, ^22^, ^23^ Although diaphragm preservation may be preferred over diaphragm resection, given its association with less peritoneal progression, improved survival, and reduced perioperative morbidity, it is not always feasible when aiming for a macroscopic complete resection.^8^ In our cohort, 52 of 70 (74%) of surgical patients required diaphragm resection and reconstruction. To mitigate complications from this more extensive operation, we used a porcine-derived acellular dermal matrix mesh. Preliminary data suggest this biologic mesh may be a viable alternative to synthetic mesh and could improve perioperative outcomes for patients with PM. This aligns with a supplementary exploratory analysis we performed, which found no statistically significant difference in overall survival by diaphragm resection status. Looking ahead, further reductions in perioperative mortality may result from minimally invasive approaches, enabled by advances in robotic techniques, or from selective incomplete resections allowing earlier initiation of adjuvant therapy.^24^, ^25^, ^26^, ^27^

Regionalization of surgery has been associated with improved outcomes for patients with PM. Our health care system's regionalized model has increased surgical use by an experienced PM surgeon and ensured guideline-based management through an expert multidisciplinary team including a mesothelioma-specialized oncologist.^17^^,^^18^ All patient cases are reviewed by a multidisciplinary PM tumor board, which enhances diagnostic accuracy, prognostic assessment, and treatment planning. If determined an appropriate operative candidate by the tumor board, patients are typically offered upfront surgery and routine adjuvant systemic therapy. The high-volume nature of our specialized program ensures all members of the perioperative team, including the operating room staff and postoperative nurses, are experienced in caring for these complex patients.^20^ It is worth noting that the early years of our cohort (2009-2013) predated the regionalization of mesothelioma care within our healthcare system. As a result, a subset of patients with early-stage disease received only systemic therapy, likely reflecting limited access to surgical expertise at the time. More recently, an increasing proportion of patients with mesothelioma have been receiving multimodal therapy.

Despite the strengths of our PM program, our 30- and 90-day mortality rates were greater than those reported in MARS2 (4% and 9%, respectively). We believe this reflects high-risk features in our cohort. For example, among the 3 patients in our “favorable-risk” early-stage epithelioid subgroup who died within 90 days, one had severe comorbidity (CCI ≥7). This illustrates the importance of considering the full spectrum of patient characteristics in treatment planning, even when patients otherwise meet clinical criteria permitting PD. Furthermore, the concentration of early postoperative deaths in the overall cohort among patients with advanced disease stages, mixed histology, and significant comorbidity suggests that, for these greater-risk patients in particular, PD may shorten survival and should therefore be discouraged.

### Limitations

Our study has several limitations. First, as a retrospective analysis, it is subject to selection bias and lacks an intent-to-treat variable. Treatment groups reflect therapies received, not the initially intended treatments. Although we adjusted for known variables, residual confounding is likely because many factors (eg, frailty status) potentially influencing treatment selection were unavailable. As a descriptive study, comparisons between treatment groups are not intended to establish causation. Second, our study did not distinguish patients by specific systemic treatment regimens, including receipt of immunotherapy. Although there is an increasing number of patients receiving immunotherapy, its role in PM treatment continues to evolve and the number of patients receiving immunotherapy remains small.^7^^,^^17^ Furthermore, despite anticipated changes in systemic therapy over the study period, diagnosis year was not a significant predictor of mortality. This lack of a temporal effect suggests that grouping systemic therapies likely did not obscure a meaningful survival difference due to potential shifts in systemic treatment patterns. Lastly, because our data were derived from patients treated within the KPNC system, generalizability to other hospitals may be limited. However, given the complexity of PM surgery and the need for care at high-volume centers with expert multidisciplinary teams, we believe KPNC provides an ideal setting to study outcomes in PM. In doing so, our findings may also help promote a model of specialized PM care for other institutions.

## Conclusions

In this real-word cohort of patients with PM, multimodal therapy incorporating both surgery and systemic treatment was associated with significantly improved OS, specifically among an early-stage epithelioid subgroup. These findings support the continued role of surgery in PM but underscore that it must be performed in the right patients and with the right approach. Furthermore, high-level prospective research is still needed. As more effective systemic options, including immunotherapy, emerge and targeted radiation protocols are adopted, understanding how surgery may act synergistically with these modalities will be critical to improve outcomes for patients with PM.

## Conflict of Interest Statement

The authors reported no conflicts of interest.

The *Journal* policy requires editors and reviewers to disclose conflicts of interest and to decline handling or reviewing manuscripts for which they may have a conflict of interest. The editors and reviewers of this article have no conflicts of interest.

## Figures and Tables

**Table E1:** Characteristics of patients with epithelioid histology (N = 226)

Characteristics	No treatment (n = 83)	Surgery (n = 16)	Systemic (n = 87)	Multimodal (n = 40)	*P* value
Age at diagnosis, y					<.0001
Mean ± SD	77.3 ± 12.2	71.4 ± 11.4	72.4 ± 8.5	68.1 ± 10.3	
Median (IQR)	80.0 (72.0-86.0)	74.5 (70.0-78.5)	73.0 (68.0-78.0)	70.0 (64.0-74.0)	
CCI					.84
0-3	33 (39.8)	5 (31.3)	41 (47.1)	18 (45.0)	
4-6	25 (30.1)	6 (37.5)	27 (31.0)	11 (27.5)	
≥7	25 (30.1)	5 (31.3)	19 (21.8)	11 (27.5)	
Sex					.37
Male	55 (66.3)	11 (68.8)	68 (78.2)	28 (70.0)	
Female	28 (33.7)	5 (31.3)	19 (21.8)	12 (30.0)	
Race/ethnicity					.50
White	54 (65.1)	10 (62.5)	69 (79.3)	26 (65.0)	
Black	5 (6.0)	2 (12.5)	3 (3.4)	1 (2.5)	
Hispanic	13 (15.7)	3 (18.8)	9 (10.3)	6 (15.0)	
Asian/Pacific Islander	6 (7.2)	0	5 (5.7)	4 (10.0)	
Native American/ Multiracial/other/unknown	5 (6.0)	1 (6.3)	1 (1.1)	3 (7.5)	
Clinical stage					.07
1	24 (28.9)	6 (37.5)	30 (34.5)	10 (25.0)	
2	16 (19.3)	4 (25.0)	12 (13.8)	10 (25.0)	
3	23 (27.7)	5 (31.3)	17 (19.5)	16 (40.0)	
4	20 (24.1)	1 (6.3)	28 (32.2)	4 (10.0)	
Smoking status					.71
Current	3 (3.6)	0	6 (6.9)	1 (2.5)	
Former	46 (55.4)	9 (56.3)	48 (55.2)	19 (47.5)	
Never	34 (41.0)	7 (43.8)	33 (37.9)	20 (50.0)	
BMI					.65
<25	38 (45.8)	7 (43.8)	32 (36.8)	15 (37.5)	
25.0-29.9	30 (36.1)	6 (37.5)	43 (49.4)	16 (40.0)	
≥30	15 (18.1)	3 (18.8)	12 (13.8)	9 (22.5)	
Diagnosis year					<.0001
2009-2014	37 (44.6)	2 (12.5)	29 (33.3)	1 (2.5)	
2015-2020	31 (37.3)	9 (56.3)	46 (52.9)	27 (67.5)	
2021-2023	15 (18.1)	5 (31.3)	12 (13.8)	12 (30.0)	

*SD*, Standard deviation; *IQR*, interquartile range; *CCI*, Charlson Comorbidity Index; *BMI*, body mass index.

**Table E2:** Characteristics of early-stage patients (N = 185)

Characteristics	No treatment (n = 84)	Surgery (n = 16)	Systemic (n = 64)	Multimodal (n = 21)	*P* value
Age at diagnosis, y					<.0001
Mean ± SD	79.4 ± 9.1	71.8 ± 9.8	73.0 ± 8.7	69.2 ± 8.6	
Median (IQR)	81.0 (75.5-86.0)	74.5 (71.0-77.5)	74.0 (66.5-79.0)	70.0 (63.0-74.0)	
CCI					.49
0-3	34 (40.5)	7 (43.8)	35 (54.7)	8 (38.1)	
4-6	28 (33.3)	3 (18.8)	16 (25.0)	6 (28.6)	
≥7	22 (26.2)	6 (37.5)	13 (20.3)	7 (33.3)	
Sex					.34
Male	62 (73.8)	12 (75.0)	52 (81.3)	13 (61.9)	
Female	22 (26.2)	4 (25.0)	12 (18.8)	8 (38.1)	
Race/ethnicity					.60
White	60 (71.4)	10 (62.5)	48 (75.0)	15 (71.4)	
Black	5 (6.0)	1 (6.3)	1 (1.6)		
Hispanic	10 (11.9)	4 (25.0)	6 (9.4)	3 (14.3)	
Asian/ Pacific Islander	7 (8.3)		4 (6.3)	1 (4.8)	
Native American/ Multiracial/other/unknown	2 (2.4)	1 (6.3)	5 (7.8)	2 (9.5)	
Clinical stage					.21
1	51 (60.7)	8 (50.0)	46 (71.9)	11 (52.4)	
2	33 (39.3)	8 (50.0)	18 (28.1)	10 (47.6)	
Histology					<.01
Epithelioid	40 (47.6)	10 (62.5)	42 (65.6)	20 (95.2)	
Biphasic	12 (14.3)	4 (25.0)	5 (7.8)	1 (4.8)	
Sarcomatoid	9 (10.7)	1 (6.3)	6 (9.4)	0	
NOS	23 (27.4)	1 (6.3)	11 (17.2)	0	
Smoking status					.39
Current	2 (2.4)	1 (6.3)	5 (7.8)	0	
Former	49 (58.3)	7 (43.8)	34 (53.1)	9 (42.9)	
Never	23 (27.4)	8 (50.0)	25 (39.1)	12 (57.1)	
BMI					.35
<25	44 (52.4)	5 (31.3)	24 (37.5)	8 (38.1)	
25.0-29.9	25 (29.8)	7 (43.8)	30 (46.9)	8 (38.1)	
≥30	15 (17.9)	4 (25.0)	10 (15.6)	5 (23.8)	
Diagnosis year					<.0001
2009-2014	52 (61.9)	3 (18.8)	27 (42.2)	0	
2015-2020	24 (28.6)	8 (50.0)	31 (48.4)	17 (81.0)	
2021-2023	8 (9.5)	5 (31.3)	6 (9.4)	4 (19.0)	

*SD*, Standard deviation; *IQR*, interquartile range; *CCI*, Charlson Comorbidity Index; *NOS*, not otherwise specified; *BMI*, body mass index.

**Table E3:** Cox proportional hazards model epithelioid and early-stage subgroups

Variable	Epithelioid subgroup		Early-stage subgroup	
	aHR (95% CI)	*P* value	aHR (95% CI)	*P* value
Period				
2009-2014	Reference		Reference	
2015-2020	1.09 (0.77-1.53)	.64	0.93 (0.61-1.41)	.72
2021-2023	0.74 (0.45-1.22)	.24	0.56 (0.29-1.09)	.09
Age, y				
18-64	Reference		Reference	
65-74	1.37 (0.87-2.16)	.18	1.44 (0.79-2.64)	.23
75-84	1.44 (0.91-2.26)	.12	1.71 (0.94-3.11)	.08
≥85	1.63 (0.93-2.86)	.09	2.08 (1.07-4.04)	.03
Treatment				
No treatment	Reference		Reference	
Surgery	0.72 (0.37-1.39)	.32	0.88 (0.45-1.71)	.71
Systemic	0.47 (0.33-0.67)	<.001	0.75 (0.51-1.11)	.15
Multimodal	0.25 (0.15-0.44)	<.001	0.43 (0.21-0.89)	.02
Sex				
Female	Reference		Reference	
Male	0.94 (0.68-1.30)	.70	1.05 (0.71-1.54)	.82
CCI				
0-3	Reference		Reference	
4-6	1.06 (0.74-1.51)	.75	0.84 (0.56-1.25)	.38
≥7	1.38 (0.95-2.00)	.09	1.27 (0.83-1.93)	.27
Histology				
Nonepithelioid	–		Reference	
Epithelioid	–		0.62 (0.43-0.88)	<.01
Stage				
I-II	0.51 (0.38-0.70)	<.001	–	
III-IV	Reference		–	

*aHR*, Adjusted hazards ratio; *CCI*, Charlson Comorbidity Index.

**Table E4:** Postoperative outcomes across epithelioid and early-stage subgroups

Outcomes	Epithelioid subgroup (n = 56)	Early-stage subgroup (n = 37)
30-d emergency department visit	14 (25.0)	10 (27.0)
30-d readmission	6 (10.7)	4 (10.8)
30-d postoperative complication∗	28 (50.0)	18 (48.6)
30-d major postoperative complication†	5 (8.9)	4 (10.8)
30-d mortality	3 (5.3)	3 (8.1)
90-d mortality	6 (10.7)	5 (13.5)

∗ Postoperative complications included anemia, chyle leak, hypotension, pleural effusion, wound infection, pneumonia, empyema, pulmonary embolus/deep vein thrombosis, pericardial effusion, atrial fibrillation, prolonged air leak, bronchopleural fistula, prolonged intubation, reintubation, tracheostomy, prolonged supplemental oxygen, mucous plug, atelectasis, urinary retention, urinary tract infection, stroke, acute respiratory failure, cardiac arrest, and deep vein thrombosis.

† Major complications included acute respiratory failure, cardiac arrest, or death within 30 days.
